# Association of serum BDNF levels and the BDNF Val66Met polymorphism with the sleep pattern in healthy young adults

**DOI:** 10.1371/journal.pone.0199765

**Published:** 2018-06-26

**Authors:** Kaori Saitoh, Ryuji Furihata, Yoshiyuki Kaneko, Masahiro Suzuki, Sakae Takahashi, Makoto Uchiyama

**Affiliations:** Department of Psychiatry, Nihon University School of Medicine, Tokyo, Japan; Chiba Daigaku, JAPAN

## Abstract

**Background:**

Brain-derived neurotrophic factor (BDNF) is widely expressed in the brain and plays an important role in neuronal maintenance, plasticity, and neurogenesis. Prior studies have found that decreased serum BDNF levels are associated with perceived stress, depression, or sleep disturbances in humans.

**Study objectives:**

To elucidate whether the serum BDNF levels and BDNF genotype were associated with the sleep pattern in healthy young adults.

**Methods:**

The study group consisted of 79 healthy paid volunteers (45 men, 34 women) aged 20 to 29 years. Serum BDNF levels were measured with an enzyme-linked immunosorbent assay, and a single-nucleotide polymorphism (Val66Met) in the BDNF gene was assessed with a TaqMan assay. Details of the sleep pattern were obtained from 1-week sleep/wake records.

**Results:**

Serum BDNF levels were significantly associated with sleep parameters on weekends, whereas no such association was found on weekdays. On weekends, longer total sleep time and time in bed, and later mid-sleep time were associated with lower serum BDNF levels. The difference between mid-sleep time on weekdays and that on weekends, otherwise known as social jetlag, was negatively associated with serum BDNF levels. Met/Met homozygotes of the BDNF Val66Met polymorphism had significantly longer time in bed on weekends than Val/Val homozygotes. Heterozygotes did not differ from Val/Val homozygotes.

**Conclusions:**

We first found that serum BDNF levels and the BDNF Val66Met polymorphism in healthy young adults were associated with the sleep pattern on weekends but not with that on weekdays, suggesting that the systems involved in BDNF control may be linked to endogenous sleep characteristics rather than the socially constrained sleep schedule in healthy young adults.

## Introduction

Brain-derived neurotrophic factor (BDNF), a member of the neurotrophin family, is widely expressed in the brain and the periphery, and plays an important role in neuronal maintenance, plasticity, and neurogenesis [1, 2]. Circulating levels of BDNF have been proposed as a possible marker of diseases [3–5]. BDNF levels are lower in humans with depression [6, 7], bipolar disorder [8, 9], schizophrenia [10, 11], and dementia [12] compared to age-matched controls. In addition, several studies demonstrated that successful treatment of depression [13] and bipolar disorder [14] normalizes peripheral BDNF concentrations, suggesting that BDNF is a possible marker of disease recovery. Studies on non-clinical populations demonstrated that perceived stress is negatively associated with circulating BDNF levels. A negative correlation is present between serum BDNF levels and work-related perceived stress in workers [15, 16], and romantic stress in healthy young adults [17]. Serum BDNF levels are also positively associated with stress resilience in healthy women [18]. Several reports have documented that decreased circulating BDNF levels are associated with disturbed sleep [19, 20]. An epidemiological survey revealed that serum BDNF levels are lower in women with sleep disturbances [19]. Other studies have reported that serum BDNF levels are correlated with perceived severity of insomnia [20], and polysomnographically confirmed disturbances in non-rapid eye movement sleep [21], i.e., reduced theta EEG activity and reduced percentage of deep non-rapid eye movement sleep. These studies imply that BDNF may participate in homeostatic regulation of sleep that controls brain recovery.

These previous studies have shown that circulating BDNF may be a state-dependent marker in patients with psychiatric disorders, an objective marker of psychological stress in healthy subjects, and a potential indicator of insufficient sleep and a consequence of poor brain recovery in healthy subjects. More recently, an observational study from Switzerland examined the relationship among stress, sleep, and BDNF levels and suggested an interaction between BDNF levels and sleep [22], which plays an essential role in brain recovery. This relationship may also provide an explanation for decreased BDNF levels in subjects complaining of perceived stress and levels in patients with stress-related mental disorders, because sleep is commonly disturbed both in stressful conditions and most mental disorders [23]. However, a fundamental understanding of the physiological interaction between BDNF levels and sleep in healthy humans remains limited, although a relationship between BDNF levels and several types of sleep pathology have been reported. Here we studied for the first time the relationship between the sleep pattern and serum BDNF levels, and between the sleep pattern and BDNF genotypes in healthy young subjects. Based on previous findings of an age-related decline in BDNF levels [24, 25], we employed healthy young adults within a narrow age range (20 to 40 years) to exclude the confounding effects of age in the present study.

## Methods

### Study subjects and data collection

Subjects were recruited via flyers posted on the Nihon University bulletin board from July 2015 to October 2015. A total of 103 individuals applied to participate in the study. Participants were selected by psychiatric specialists (KS and MU) using the following criteria: 1) those aged 20 to 40 years, 2) those who did not engage in shift or midnight work, 3) those who were free from social dysfunction, 4) those who did not have a history of sleep, neurological, or psychiatric disorders, or any history of using psychoactive drugs, and 5) those whose score on the Japanese version of the Center for Epidemiologic Studies Depression Scale [26] was less than 16 points.

Finally, we recruited 79 healthy paid volunteers (45 men and 34 women) aged 20 to 29 years in the study, and excluded 24 for the following reasons: age >40 years (n = 5); shift work (n = 5); social dysfunction (n = 1); psychoactive drugs (n = 2); Center for Epidemiologic Studies Depression Scale scores ≥16 (n = 16). The participants were medical students or doctors, and no one had type 2 diabetes, which potentially influences serum BDNF levels [27].

The study was comprised of three parts: (1) evaluation of the sleep pattern based on sleep/wake records and a questionnaire, (2) measurement of serum BDNF concentration, and (3) genotyping of a BDNF polymorphism. All the sleep data and blood samples were obtained within 10 days. The study was approved by the local ethics committee of Nihon University School of Medicine. Written informed consent was obtained from all individuals who applied to participate in the study.

#### Sleep/Wake records

The participants were instructed to record 1) bed time, 2) wake-up time, and 3) sleep onset latency every day for 7 days or more. Sleep onset time, time in bed (TIB), total sleep time (TST), and mid-sleep time (MS) were calculated from 1)-3) and were averaged separately for weekdays and weekends. We further calculated social jetlag as defined below.

Sleep onset time: bed time − sleep onset latency

TIB: time from bed time to wake-up time

TST: time from sleep onset time to wake-up time

MS: mid-point of sleep onset time and wake-up time

Social jetlag: mean MS of weekends–mean MS of weekdays

#### Questionnaire

We obtained the participant’s body weight, height, alcohol consumption, and smoking habits from self-reported information. We defined subjects who had smoked more than 100 cigarettes for over 6 months as habitual smokers, and those who drank over 22 g pure alcohol for more than three times a week as habitual drinkers.

Sleep quality and sleep problems were measured using the Japanese version of the Pittsburgh Sleep Quality Index (PSQI-J) [28]. PSQI assesses subjective sleep quality, sleep latency, sleep duration, habitual sleep efficiency, sleep disturbances, use of sleeping medication, and daytime dysfunction over the course of 1 month. Subjects with higher total scores have more serious sleep disorders.

#### Blood investigations

Serum BDNF examination. We collected blood between 7:00 and 8:30 AM before breakfast and centrifuged the samples, which were stored at −80°C. We measured the BDNF concentration with an enzyme-linked immunosorbent assay using a Quantikine®ELISA kit (R&D Systems, Minneapolis, MN, USA).

BDNF polymorphism. Genomic DNA was extracted from peripheral blood leukocytes using the Genomic DNA extraction kit (TALENT, Trieste, Italy). Genotyping of the single-nucleotide polymorphism was performed using TaqMan technology on the Applied Biosystems 7500 Fast Real-Time PCR system (Applied Biosystems, Foster City, CA, USA).

### Statistical analyses

The presence of gender difference was examined with respect to age, body mass index (BMI), smoking, alcohol, parameters measured in the sleep/wake records, and serum BDNF with the *t*-test and χ^2^test. We first examined the distribution of the serum BDNF data with the Kolmogorov-Smirnov test, and the Pearson test was performed to assess the correlations between serum BDNF and other factors (age, BMI, smoking, alcohol, and parameters measured in the sleep/wake records). The associations between the Val66Met polymorphism and sleep pattern were examined with the *t*-test. The level of significance was set at p < 0.05. All statistical analyses were conducted with SPSS ver. 21.0.

## Results

Demographic features, serum BDNF levels, and sleep variables of the participants are shown in Table 1. The Kolmogorov-Smirnov test revealed that serum BDNF levels were normally distributed. Allele distributions followed Hardy-Weinberg equilibrium (χ^2^ = 2.58; p = 0.89). Genotype frequencies of 78 subjects were Val/Val 0.36 (29/78), Val/Met 0.45 (35/78), and Met/Met 0.18 (14/78). We did not find any significant differences in age (F = 0.1, p = 0.90) or sex (χ^2^ = 1.29, p = 0.53) among the three genotype groups.

**Table 1:** Characteristics of study participants.

	average	SD
Age (years)	24	1.94
Sex (M:F)	45:34	
BMI	21.54	3.07
Smoking	8.9%	
Drinking alcoholic beverages	27.8%	
Serum BDNF (pg/mL)	26060	5814
【Sleep variables】		
PSQI	5.09	2.34
Wake-up time weekdays (h:min)	7:22	1:03
Wake-up time weekends (h:min)	8:16	1:39
TST weekdays (h)	6.42	1.38
TST weekends (h)	6.94	1.17
TIB weekdays (h)	6.70	1.35
TIB weekends (h)	7.16	1.17
MS weekdays (h:min)	3:26	0:48
MS weekends (h:min)	3:40	0:40
Social jetlag (h)	0.22	0.96

SD: standard deviation, BMI: body mass index, PSQI: the Pittsburgh Sleep Quality Index, TST: total sleep time, TIB: time in bed, MS: mid-sleep time.

The statistical comparison of wake up time, sleep onset time, TST, TIB, and MST with respect to weekdays and weekends is shown in Table 2. These sleep parameters differed significantly between weekdays and weekends.

**Table 2:** The differences in sleep parameters between weekdays and weekends.

	Weekdays	Weekends	p value
Sleep onset time (h:min)	0:57	1:20	0.041
TIB (h)	6.70	7.16	0.015
TST (h)	6.42	6.94	0.007
MS (h:min)	3:26	3:40	0.046
Wake-up time (h:min)	7:22	8:16	0.00002

TST: total sleep time, TIB: time in bed, MS: mid-sleep time.

**Fig 1:**
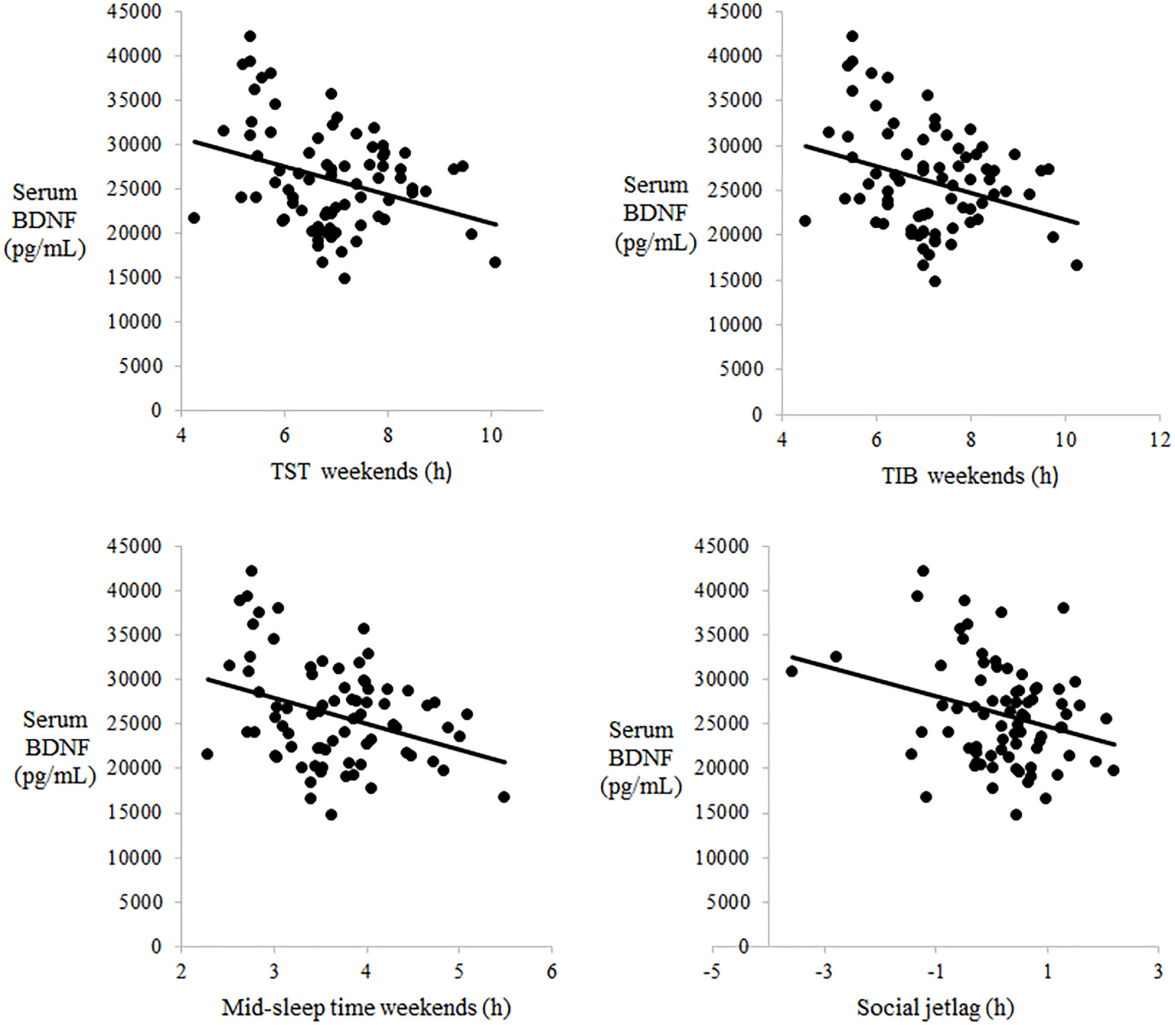
Correlations between serum BDNF levels and the sleep pattern on weekends. Analyses showed significant correlations between serum BDNF levels and the sleep pattern: TST on weekends (r = −0.32, p < 0.01), TIB on weekends (r = −0.30, p < 0.01), MS on weekends (r = −0.33, p < 0.01), and social jetlag (r = −0.28, p < 0.05).

Correlation analyses between serum BDNF levels and age, BMI, smoking habits, alcohol habits, and the PSQI score did not reveal any significant differences (Table 3). Serum BDNF levels were not correlated with any sleep parameters on weekdays, whereas the levels were significantly correlated with TIB on weekends (r = −0.30, p < 0.01), TST on weekends (r = −0.32, p < 0.01), MS on weekends (r = −0.33, p < 0.01), and social jetlag (r = −0.28, p < 0.05) (Fig 1). Distribution patterns and regression lines of parameters showing a significant correlation are presented in Fig 1.

**Table 3:** Serum BDNF levels correlate with the sleep pattern on weekends.

	r	p-value
Age (years)	0.15	0.19
BMI	−0.04	0.71
Smoking		0.80
Drinking alcohol beverages		0.51
PSQI	−0.04	0.72
Wake-up time weekdays	0.10	0.37
Wake-up time weekends	0.00	0.99
Sleep onset time weekdays	0.11	0.35
Sleep onset time weekends	0.21	0.06
TST weekdays	−0.02	0.84
TST weekends	−0.32	0.01
TIB weekdays	0.03	0.82
TIB weekends	−0.30	0.01
MS weekdays	0.06	0.58
MS weekends	−0.33	0.003
Social jetlag	−0.28	0.01

BMI: body mass index, PSQI: the Pittsburgh Sleep Quality Index, TST: total sleep time, TIB: time in bed, MS: mid-sleep time.

**Fig 2:**
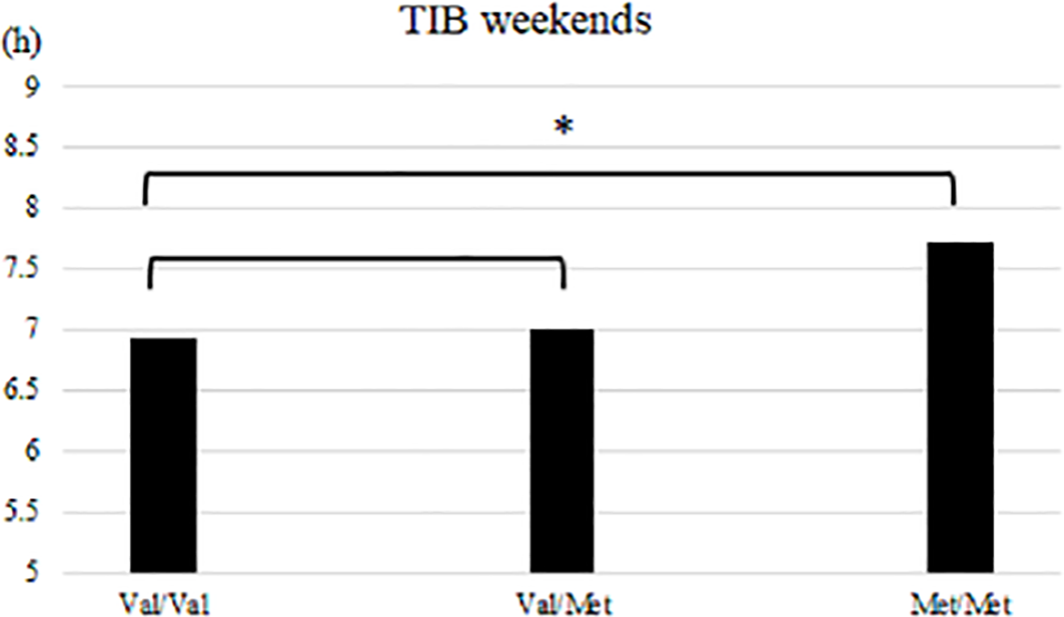
A significant difference was found for TIB on weekends between Val homozygotes and Met homozygotes.

In terms of sleep variables either on weekdays or weekends, the Val homozygotes and heterozygotes did not differ significantly, nor did Val homozygotes and Met homozygotes except for TIB on weekends, which was nearly 1 hour shorter in Val homozygotes than in Met homozygotes (Table 4, Fig 2).

**Table 4:** Comparison of the BDNF Val/Val (n = 29), Val/Met (n = 35), and Met/Met (n = 14) genotypes.

	Genotype			p-value	
	V/V	V/M	M/M	V/V vs. V/M	V/V vs. M/M
PSQI	5.00	5.00	4.50	0.84	0.37
Wake-up time weekdays (h:min)	7:00	7:30	6:54	0.25	0.74
Wake-up time weekends (h:min)	7:45	8:00	8:17	0.48	0.20
Sleep onset time weekdays (h:min)	0:53	1:06	1:07	0.58	0.86
Sleep onset time weekends (h:min)	1:10	1:10	0:42	0.84	0.56
TST weekdays (h)	6.50	6.25	6.50	0.61	0.44
TST weekends (h)	6.82	6.83	7.33	0.62	0.06
TIB weekdays (h)	6.50	6.60	6.67	0.65	0.51
TIB weekends (h)	6.92	7.00	7.71	0.38	0.04
MS weekdays (h:min)	3:23	3:20	3:23	0.91	0.78
MS weekends (h:min)	3:32	3:31	3:43	0.76	0.21
Social jet lag (h)	0.19	0.44	0.43	0.88	0.52

Analyses showed a significant difference in TIB on weekends between Val/Val and Met/Met. V/V: Val/Val, V/M: Val/Met, M/M: Met/Met, PSQI: the Pittsburgh Sleep Quality Index, TST: total sleep time, TIB: time in bed, MS: mid-sleep time.

## Discussion

This is the first study to investigate the relationships of serum BDNF levels and BDNF genotype with sleep habits such as chronotype and social jetlag in healthy subjects. The key findings were 1) serum BDNF levels were significantly correlated with sleep parameters, including TST on weekends, TIB on weekends, MS on weekends, and social jetlag, and 2) Met/Met homozygotes showed significantly longer time in bed on weekends than Val/Val homozygotes.

Previous reports have pointed out that, in modern society, one’s sleep timing on weekdays is mainly determined by school, work, and/or social constraints irrespective of the internal circadian clock, whereas on weekends, fewer constraints permit one to sleep on a more preferable timing according to the internal circadian clock [29, 30]. Therefore, sleep timing on weekends or free days has been regarded as a variable that represents the timing of the endogenous circadian rhythm. The MS on weekends, which Zavada et al. [29] first proposed, has been used as a circadian phase marker that correlates well with differences in chronotype as measured with a morningness-eveningness questionnaire [31]; early and late MS on weekends indicate morningness and eveningness, respectively. The present results showing that MS on weekdays was not correlated with serum BDNF levels but that MS on weekends was negatively correlated with serum BDNF levels in healthy young adults may indicate that morningness or eveningness was associated with increased or decreased serum BDNF levels. These results also provide the first documentation of the relationship between chronotype and BDNF.

Prior epidemiological surveys have reported that TST on weekends is longer than that on weekdays in industrialized countries [32, 33], and the authors postulated that the discrepancy is due to sleep insufficiency on weekdays and homeostatic sleep compensation on weekends [34–36]. We found that TST and TIB on weekends showed negative correlations with serum BDNF levels, whereas those on weekdays did not. In the present study, the explanation for sleep prolongation on weekends seemed to include two possibilities: compensatory sleep prolongation for sleep debt on weekdays or natural manifestation of a longer sleep tendency with fewer social constraints on weekends. Our present results about the relationship between BDNF and TIB or TST may be interpreted according to these two possibilities.

Social jetlag is defined as the difference between one’s MS on weekdays and that on weekends, indicating the degree of sleep misalignment with the endogenous circadian rhythm on weekdays, which is estimated to account for some psychosomatic difficulties on weekdays, especially on Monday mornings [37]. We found that social jetlag in the present subjects was negatively correlated with serum BDNF levels. Given that social jetlag is associated with consequences due to misalignment between the actual sleep schedule and the endogenous circadian rhythm, BDNF levels may be associated with certain subjective difficulties possibly related to sleep misalignment [37]. However, we did not find any correlation between serum BDNF levels and the PSQI score. Accordingly, further studies should focus on the relationship between BDNF and subjective consequences potentially due to such misalignment.

The present analysis of the BDNF Val66Met polymorphism among healthy young subjects demonstrated that TIB on weekends was significantly longer in Met homozygotes than in Val homozygotes. A similar non-significant tendency was found in TST on weekends, but no such differences were found in sleep parameters on weekdays. These results suggest that the BDNF Val66Met polymorphism had an influence on endogenous sleep characteristics of the subjects. This is the first documentation of a possible link between the BDNF Val66Met polymorphism and endogenous sleep characteristics. Other associated features of the BDNF Val66Met polymorphism include a potential brain morphological difference in the developmental period [38]. Hashimoto et al. [38] reported that the volumes of the right cuneus, left insula, and left ventromedial prefrontal cortex are different between BDNF Met homozygotes and Val homozygotes in adolescents, providing evidence that the Val66Met polymorphism may influence the volume of the human brain. We postulate that some morphological differences in the brain affect sleep characteristics. Further studies are needed to examine the relationship.

Mechanisms of the observed relationship between serum BDNF levels and the sleep pattern on weekends were not clarified in the present study. However, several studies have provided information about the mechanisms [39, 40]. Chronic sleep loss is associated with increased cortisol levels, especially around habitual bedtime [39]. An animal study suggested that cortisol affects BDNF levels in the brain [40]. Further study is needed to investigate the role of cortisol in the association between serum BDNF levels and the sleep pattern observed in the present study.

### Limitations

This study has several limitations. First, this was a cross-sectional study, and we could not determine a causal relationship. A further interventional study is needed to clarify a possible causal relationship. Second, we measured the sleep pattern with sleep/wake records, which is a subjective type of assessment. Moreover, we did not evaluate daytime sleep or sleepiness in the participants. The observations must be confirmed with an objective instrument, such as an actigraph or polysomnography, and evaluation of daytime sleep or sleepiness, to obtain more precise results. Third, despite including 79 healthy subjects, this sample size was relatively small for obtaining a clear relationship between the sleep pattern and serum BDNF levels including the BDNF polymorphism. Furthermore, we enrolled subjects within a very narrow age range (20 to 40 years). To increase the generalizability of these findings, future research in a larger sample size with a wider age range will be required. Finally, we measured serum levels of BDNF using the Quantikine®ELISA kit (R&D Systems, Minneapolis, MN, USA), which is commonly used worldwide. This kit measures total BDNF including mature BDNF and its precursor protein proBDNF. Although mature BDNF is synthesized from proBDNF, recent studies have reported that proBDNF and mature BDNF elicit opposing effects via the p75^NTR^ and TrkM receptors, respectively [41]. Yoshida et al. reported decreased levels of mature BDNF, but not proBDNF, in major depression [42]. A strong correlation (r = 0.701) was reported between serum levels of total BDNF using the R&D Systems kit and those of mature BDNF using the kit from Adipo Bioscience [43]; however, our findings should be confirmed in future studies by simultaneously measuring mature BDNF levels and proBDNF.

## Conclusions

In conclusion, these results suggested that systems involved in BDNF control may be linked to endogenous sleep characteristics rather than the socially constrained sleep schedule in healthy young subjects.
