# Correction: Association of serum BDNF levels and the BDNF Val66Met polymorphism with the sleep pattern in healthy young adults

**DOI:** 10.1371/journal.pone.0201994

**Published:** 2018-08-02

**Authors:** Kaori Saitoh, Ryuji Furihata, Yoshiyuki Kaneko, Masahiro Suzuki, Sakae Takahashi, Makoto Uchiyama

The sixth author, Makoto Uchiyama, is incorrectly listed as the corresponding author. The correct corresponding author is Masahiro Suzuki. Masahiro Suzuki’s email address is suzuki.masahiro94@nihon-u.ac.jp.
